# Sex in Education

**Published:** 1874-02

**Authors:** 


*Sex in Education; or a Fair Chance for the Girls.* By Edward H. Clarke, M. D., etc. Boston: James R. Osgood & Co., 1873. Buffalo: Martin Taylor.

This little book is the outgrowth of an essay which the author read about a year since before the Women's Club of Boston. The ideas of the author at that time attracted considerable attention and so many were the inquiries addressed to him concerning his views, that he was induced to review the whole subject carefully, and the result has been, the book now before us. The topic under consideration is one of general interest and it is discussed in a manner which shows careful study and a desire to place the subject in its true light. Now that the subject of female education is and has been attracting so much attention, we are glad to see that Dr. Clarke has seen the necessity of looking at the matter from a physiological point of view. It is from this stand point that the subject is in the future to be decided, and the author has opened the discussion in a manner which will carry conviction to many of his readers.

Those who have hitherto insisted that the girl was capable of going through with the same course of study as her brother in the same manner and space of time have overlooked the physiological differences which distinguish her from the male sex. It is against this viscious system that this book is directed. Dr. Clarke has had opportunities of observing the working of this system and its results and the arguments which he brings against it are supported by sound medical judgment. He speaks plainly, and does not endeavor to cover up the truth or obscure his meaning by the use of obscure and blind phrases. Written by a physician of experience, it will be read by his professional brethren with interest, but at the same time the general public will do well to read and profit by its teachings. The law which governs the education of the boy and the life of the man is continuous; it is not during any time divided into periods during which any different rules of living are found necessary.

With the girl on the contrary, after a certain age life is a system of periods. It is during the time that this change is about to take place that the young girl is most frequently made to undergo the most severe strain at school and seminary. Nor is any calculation made in the system of study which they are made to undergo that at regularly recurring monthly periods, there occurs a number of days when nature demands, and should have in order to the proper performance of a peculiar and important function, complete rest.

As a statement showing the position the physician occupies in relation to this problem, we copy from page 61: * * "The sick chamber, not the school room; the physician's private consultation, not the committee's public examination; the hospital not the college, the work shop, or the parlor, disclose the sad result which modern social customs, modern education and modern ways of labor have entailed on women. Examples of them may be found in every walk of life. On the luxurious couches of Beacon Street; in the palaces of Fifth Avenue; among the classes of our private, common and normal schools; among the female graduates of our colleges; behind the counters of Washington Street and Broadway; in our factories, workshops and homes—may be found numberless pale weak neuralgic dyspeptic, hysterical monorrhagic, dysmenorrhœic girls and women, that are living illustrations of the truth of this brief monograph. It is not asserted here that improper methods of study, and a disregard of the reproductive apparatus and its functions during the educational life of girls are the sole causes of female diseases; neither is it asserted that all female graduates of our schools and colleges are pathological specimines. But it is asserted that the number of these graduates who have been permanently disabled to a greater or less degree by these causes is so great, as to excite the gravest alarm, and to demand the serious attention of the community." We should be pleased to quote many other passages but can only say to our readers get the book study and read it for yourselves. The author has been sharply criticized by some who seem to think that in some passage they detect a thrust at some pet institution. If they do not come under the list of those who would pay no regard to the physical peculiarities of the female let them strive to bring others into a like manner of thinking rather than find fault with Dr. Clarke in the excellent work which he has undertaken.

